# Ultrafast Excited-State Decay Mechanisms of 6-Thioguanine Followed by Sub-20 fs UV Transient Absorption Spectroscopy

**DOI:** 10.3390/molecules27041200

**Published:** 2022-02-10

**Authors:** Danielle C. Teles-Ferreira, Cristian Manzoni, Lara Martínez-Fernández, Giulio Cerullo, Ana Maria de Paula, Rocío Borrego-Varillas

**Affiliations:** 1 Instituto Federal de Minas Gerais, Campus Ouro Preto, Ouro Preto 35400-000, MG, Brazil; danielle.teles@ifmg.edu.br; 2 Istituto di Fotonica e Nanotecnologie-Consiglio Nazionale delle Ricerche (IFN-CNR), Piazza Leonardo da Vinci 32, I-20133 Milano, Italy; cristian.manzoni@polimi.it (C.M.); giulio.cerullo@polimi.it (G.C.); 3 Departamento de Química, Facultad de Ciencias and Institute for Advanced Research in Chemistry (IADCHEM), Campus de Excelencia UAM-CSIC, Universidad Autónoma de Madrid, Cantoblanco, 28049 Madrid, Spain; lara.martinez@uam.es; 4 Dipartimento di Fisica, Politecnico di Milano, Piazza Leonardo da Vinci 32, I-20133 Milano, Italy; 5 Departamento de Física, Universidade Federal de Minas Gerais, Belo Horizonte 31270-901, MG, Brazil; ana@fisica.ufmg.br

**Keywords:** ultrafast spectroscopy, thiobases, global analysis

## Abstract

Understanding the primary steps following UV photoexcitation in sulphur-substituted DNA bases (thiobases) is fundamental for developing new phototherapeutic drugs. However, the investigation of the excited-state dynamics in sub-100 fs time scales has been elusive until now due to technical challenges. Here, we track the ultrafast decay mechanisms that lead to the electron trapping in the triplet manifold for 6-thioguanine in an aqueous solution, using broadband transient absorption spectroscopy with a sub-20 fs temporal resolution. We obtain experimental evidence of the fast internal conversion from the S_2_(ππ*) to the S_1_(nπ*) states, which takes place in about 80 fs and demonstrates that the S_1_(nπ*) state acts as a doorway to the triplet population in 522 fs. Our results are supported by MS-CASPT2 calculations, predicting a planar S_2_(ππ*) pseudo-minimum in agreement with the stimulated emission signal observed in the experiment.

## 1. Introduction

In 1950, George Hitchings and Gertrude Elion [1] obtained, by exchanging oxygen with sulphur in nucleobases, a potent drug for the treatment of numerous diseases [2,3,4,5]. This discovery led them to be honoured with the Nobel Prize in 1988 in the category of Physiology and Medicine. Such nucleobase derivatives are known as thiobases, and there has been a great effort to unravel their energy deactivation pathways [6,7]. The sulphur substitution in the nucleobases causes noticeable changes in the photophysics of these molecules [8,9,10,11,12,13,14,15,16,17,18,19] in comparison with the canonical nucleobases. There is a redshift of the absorption spectrum and a loss of photostability when excited by ultraviolet (UV) light. Unlike the canonical bases, which quickly repopulate the ground state after UV photoexcitation via an internal conversion (IC) process mediated by conical intersections (CIs) [20,21], the thiobases population mostly decays to a long-lived triplet state with a high quantum yield via an ultrafast intersystem crossing (ISC) process [12,22,23,24].

Due to the high transfer efficiency to the triplet states and, consequently, the high rate of singlet oxygen formation, several applications of the thiobases have been demonstrated. They are used as chemotherapeutic agents for the treatment of some types of skin cancer, as they enhance the polymerase chain reaction for DNA/RNA replication, they are used as chromophores for investigating specific interactions between nucleic acids and proteins [25,26,27,28,29,30,31,32,33]. In addition to their practical applications, they provide an excellent model system for understanding how a single atom substitution can modify the energy relaxation processes of canonical DNA/RNA nucleobases.

One of the open questions in the ultrafast dynamics of thiobases concerns the mechanism behind the efficient triplet manifold formation by ISC, which occurs shortly after the bright, excited singlet state is populated. In the literature, at least two hypotheses have been proposed: (i) the population of the triplet states mediated by a dark singlet state [12,18,22,34,35,36,37] and (ii) the direct transition from the bright singlet state to the triplet states [12,18,22,23,24,34,35,36,38]. We have recently observed a third possibility: for water-solvated thiouracils, the triplet manifold is accessed by parallel channels. While part of the population decays directly from the photoexcited bright state, another part decays passing through a dark singlet state [36,39].

In the last few years, the process of triplet state formation by ISC in 6-thioguanine (6TG) has received considerable attention in the literature [6,18,23,40,41,42,43,44,45]. Time-resolved transient absorption and photoelectron spectroscopies have been applied both in solution and gas phase for the investigation of the ultrafast dynamics in time scales, ranging from hundreds of femtoseconds up to nanoseconds. However, the primary steps after UV photoexcitation are not experimentally documented, since technical challenges have long prevented access to the sub-100 fs time scale, a highly desired benchmark as these mechanisms determine the system response on longer time scales.

In this work, we investigate the ultrafast dynamics of buffered, solvated 6TG by means of broadband transient absorption spectroscopy (TAS) with sub-20 fs UV pump pulses. Thanks to our high temporal resolution, which exceeds by a one-order magnitude that of previous studies on this molecule, and the broad spectral probing window which extends from the UV to the visible range, we are able to obtain mechanistic insight regarding the ultrafast primary processes that lead to the formation of the triplet manifold and the time constants associated with it. In particular, we show that the internal conversion from S_2_(ππ*) to the S_1_(nπ*) state takes place in about 80 fs, with the S_1_(nπ*) acting as a doorway to the triplet population in approximately 500 fs. Therefore, our results demonstrate that the decay channel S_2_(ππ*)→S_1_(nπ*)→T_1_(ππ*), experimentally observed so far only in thiouracils [36,39], is more general and can also be found in purine thiobases.

## 2. Results and Discussion

**Figure 1:**
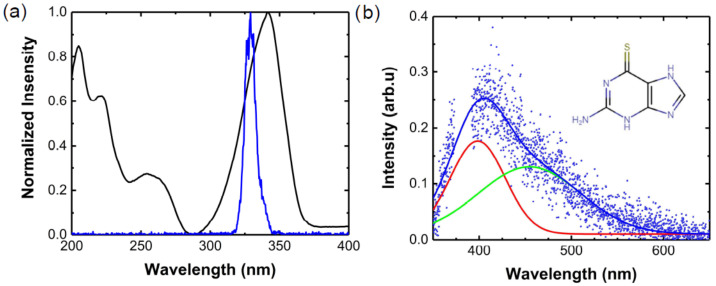
(**a**) 6TG normalised linear absorption spectrum in a phosphate saline buffer (PSB) solution at pH 7.4 (black curve) and normalised spectrum of the pump pulse employed for the TAS experiments (blue curve). (**b**) 6TG photoluminescence in a PBS solution (dots) and chemical structure of 6TG (inset). The fit (blue line) is decomposed into two Gaussians representing the contributions from the phosphorescence (green curve) and fluorescence emissions (red curve).

Figure 1a shows the 6TG steady-state linear absorption spectrum (black) and the pump pulse spectrum used for the TAS measurements (blue), while Figure 1b displays the emission spectrum. The lowest energy absorption band of 6TG presents a maximum at 340 nm (Figure 1a, black curve). Several studies [43,46,47] have assigned this band to the S_0_(ππ*)→S_2_(ππ*) transition, which is the optically active state populated by the sub-20 fs pump pulse in TAS experiments.

Figure 1b shows the steady-state photoluminescence (PL) spectra of buffered, solvated, and N_2_-pumped 6TG excited by a lamp at 340 nm. We observe a broad band that extends from 350 nm to 550 nm. Our PL spectrum is in line with the observations of Zou et al. [16]. In contrast, Reichardt et al., did not identify emission from 6TG within the sensitivity of their spectrometer [23]. Ashwood et al., observed two bands for an emission measurement at 77 K in a tris buffer 7.4 pH matrix: a weak one from 350 nm to 420 nm and another intense one from 430 nm to 550 nm [43]. The authors assigned the first band (350–420 nm, peaking at 385 nm) to the fluorescence emission. The intense band from 430 nm to 550 nm was attributed to the phosphorescence emission, which peaks at 480 nm, in reasonable agreement with the vertical excitation energy calculated for the T_1_(^3^ππ*) state at 454 nm [45] in water. As we show data at room temperature and the sample was pumped with N_2_ (in order to avoid the formation of singlet oxygen and photoluminescence quenching), we expect an overlap of the contributions from phosphorescence and fluorescence. A decomposition of the PL spectrum reveals two Gaussians centred at 398 nm (red curve in Figure 1b) and 454 nm (green curve in Figure 1b). It should be noted that there is a gap in the data from 374 to 393 nm due to a subtraction of a scattering peak from the excitation lamp. Table 1 reports the energies and oscillator strengths of 6TG in vacuum calculated at the MS-CASPT2//CASSCF(14,12)/ANO-L level of theory [48,49,50,51,52]. P1, P2, and P3 (coordinates reported in the Supplementary Materials, Table S1) are three single points along the minimum energy path relaxing the S_2_(ππ*) state from the Franck–Condon region (Supplementary Materials, Figure S1). Their close adiabatic energies suggest a planar S_2_(ππ*) pseudo-minimum. Since the maximum of the red curve in Figure 1b matches the emission wavelength calculated for this pseudo-minimum, we assign it to the fluorescence emission from the S_2_(ππ*) state. The peak of the green curve is instead attributed to the phosphorescence from the T_1_(^3^ππ*) state, as also predicted by previous works [7,43,53].

**Figure 2:**
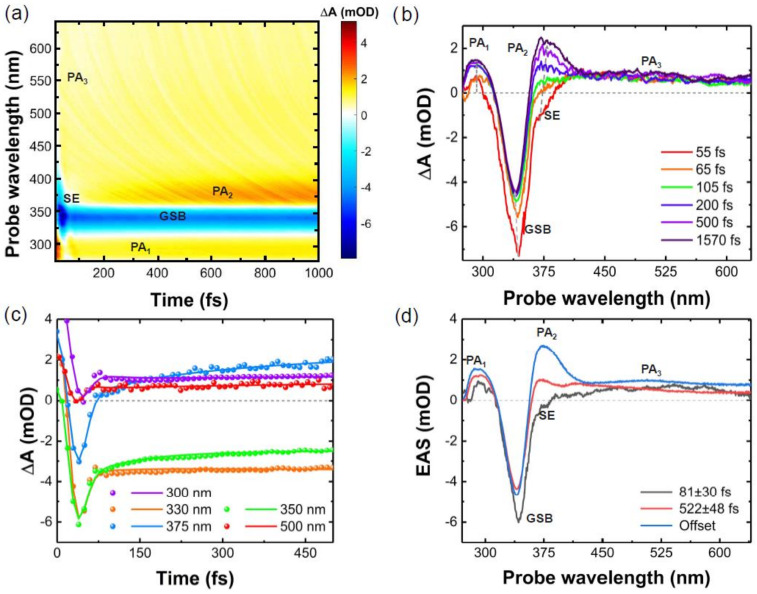
(**a**) TAS map of solvated 6TG as a function of the pump–probe delay when excited by a 330-nm pulse; (**b**) transient spectra at the time delays indicated in the legend; (**c**) dynamics at selected wavelengths during the first 500 fs. (**d**) Evolution associated spectra (EAS) obtained after the analysis of the TAS map shown in Panel (**a**).

**Figure 3:**
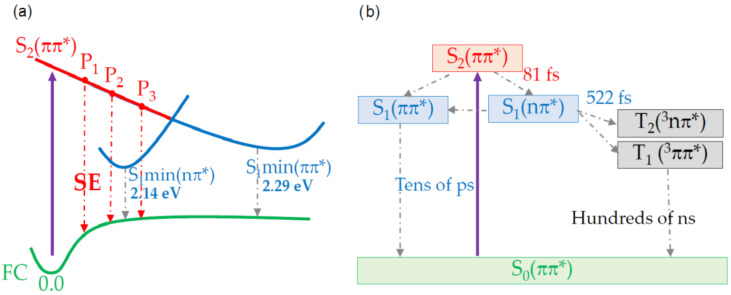
Possible 6TG triplet formation mechanism upon UV photoexcitation. (**a**) Scheme of the potential energy curves in the vicinity of the S_2_(ππ*)/S_1_(nπ*) CI: a UV photon (purple arrow) excites the S_2_(ππ*) state (yellow). Relaxation of the wave packet from S_2_(ππ*) to S_1_(nπ*) (blue) is mediated by a CI. (**b**) IC from S_2_(ππ*) to S_1_(nπ*) occurs in tens of femtoseconds as predicted in [18]. From here, several decay pathways are open: population of S_1_(ππ*), which decays by IC to the ground state in tens of picoseconds [43], and ISC to the triplets in 520 fs from S_1_(nπ*).

We performed broadband TAS experiments for 6TG in PBS solution by pumping it with sub-20 fs UV pulses centred at 330 nm and probing in a broad spectral window from 275 to 630 nm. The acquired TAS map and the transient spectra at selected time delays are displayed in Figure 2a,b, respectively, while Panel c shows the dynamics at selected wavelengths. An intense negative band peaking at 340 nm is partially superimposed with the lowest-energy band observed in the steady-state linear absorption spectra (Figure 1a), and is therefore assigned to ground state bleaching (GSB). At early times (<100 fs), we observed a negative band at about 375 nm, which is assigned to stimulated emission (SE) from the S_2_(ππ*) state. The peak of the SE band at 375 nm is in satisfactory agreement with the vertical emission energy calculated from single points at S_2_(ππ*) (P_1_, P_2_, and P_3_ in Figure 3a and Table 1) in vacuum. The estimated SE wavelength is also in line with the broadband fluorescence emission shown in Figure 1b, taking into account the experimental uncertainties of determining the position of the peaks due to the overlapping signals. The SE band completely decays in less than 80 fs, leaving two photoinduced absorption (PA) bands, PA_1_ peaking at 300 nm and the broader PA_3_ peaking about 520 nm.

At later times (>200 fs), we observed the rise of another PA band peaking at ≈375 nm (PA_2_), while the PA_3_ band remained substantially unchanged. Previous TAS measurements with 200 fs of temporal resolution have also shown long-lived PA bands centred at about 390 nm and 520 nm, which were identified as signatures of the triplet state based on their theoretical calculations [23,43].

To interpret our results, we review the previous computational studies on 6TG. Photoexcitation by the UV pump pulse creates a wave packet in the bright state, S_2_(ππ*). Reichardt et al., by combining quantum-chemical calculations and TAS with 200 fs pulses, suggested that for 6TG, most part of the population accesses the triplet manifold directly from the excited state, S_2_(ππ*), while a minor fraction decays by IC to the S_1_(nπ*), which, in-turn relaxes non-radiatively to the ground state in tens of picoseconds [23]. Theoretical studies based on MS-CASPT2//CASSCF [46] and TD-DFT [43,45] calculations have found two S_1_ minima: one of nπ* nature and another of ππ* character. They proposed that the population from the S_2_(ππ*) state decays via IC in a barrierless fashion in tens of femtoseconds to the S_1_(nπ*), which acts as a doorway to the triplet manifold. The S_1_(ππ*) minimum (see Figure 3a) enables a CI to the ground state of about 25% of the population [43] in tens of picoseconds [18]. From the S_1_(nπ*) minimum, there is an efficient population of the T_1_(ππ*) and the T_2_(nπ*) triplet states, which were found to be isoenergetic [18,46]. Then, the population is trapped in the bright triplet state for hundreds of nanoseconds [18,43].

The evolution-associated spectra (EAS) for each species were obtained by a global fit of the TAS data using a sequential kinetic model (Figure 2d). The best fit was obtained with two time constants (τ_1_ = 81 ± 30 fs and τ_2_ = 522 ± 48 fs) and an offset. The offset in the global analysis refers to time constants that are beyond our 2 ps temporal observation window (blue curve in Figure 2d). This EAS (blue curve in Figure 2d) peaks at 375 nm, with a broad band extending from 400 to 650 nm. These spectral signatures are typical of the long-lived triplet state, which has a lifetime estimated to be 830 ns [43]. The 81 ± 30 fs time constant is reported for the first time in this study (black curve in Figure 2d). Four signatures are distinguishable in the short-lived EAS. The main negative peak at 340 nm is assigned to the GSB, which is overlapped with the red-shifted negative signal at around 375 nm. The latter is assigned to the SE from the photoexcited state, S_2_(ππ*), for the following reasons: (i) it is in satisfactory agreement with the vertical emission energy calculated for the S_2_(ππ*) state (Table 1: P_1_, P_2_, and P_3_ single points); (ii) it matches with the broad fluorescence emission maximum at 385 nm [43], and it lies within the broadband PL (Figure 1b). Therefore, we estimate that the IC from S_2_(ππ*) to S_1_(nπ*) occurs on a time scale of 81 ± 30 fs, which is in line with theoretical calculations that predicted IC in about 50 fs [18]. The two PA bands at 300 nm and from 380 to 650 nm could be excited-state absorption signals from S_2_(ππ*) since they evolve on the same time scale of the SE, but we might not exclude a minor direct channel to the triplet manifold [18]. In addition, due to the planarity of the S_2_(ππ*) potential energy surface, there might be some part of the population trapped in the S_2_(ππ*), which decays at later times. Higher-level computations including spectroscopic signals of the corresponding electronic states are needed for an unambiguous assignment. The mismatch between τ_1_ and τ_2_ and the observation of SE from S_2_(ππ*) delivers the experimental confirmation about the involvement of the dark S_1_(nπ*) mediating the ISC process, as suggested by previous theoretical works for 6TG [18,43]. This shows that the S_1_(nπ*) state acts as a doorway to the triplet manifold, as also observed experimentally in some thiopyrimidines [36,39]. The τ_2_ time constant is assigned to the lifetime of the S_1_(nπ*) state (red curve in Figure 2d). It is the same value (within the error margin) reported by Ashwood et al., [43] (0.56 ± 0.06 ps), which was assigned to the formation of the triplet manifold.

Figure 3 summarises the proposed photoexcitation scenario for 6TG. Excitation at 340 nm generates a wave packet in the S_2_(ππ*) state, the signature for which is identified as an SE band with a time constant as fast as 81 fs in the TAS experiments. The CASPT2//CASSCF(14,12)/ANO-L calculations of the potential energy surface reveal a planar character of the S_2_(ππ*) state before reaching the CI to the S_1_ state, which acts as a doorway to access the triplet manifold in 522 fs. Our findings therefore provide experimental evidence of the involvement of the intermediate dark singlet state and highlight S_2_(ππ*)→S_1_(nπ*)→T_1_(ππ*) as the major decay pathway in 6TG.

## 3. Materials and Methods

### 3.1. Sample Preparation

6TG was purchased from Sigma-Aldrich (Sigma-Aldrich Chemie GmbH, Taufkirchen, Germany) with 98% purity. PBS solution was prepared by dissolving 0.15 g of monosodium phosphate and 0.27 g of sodium disodium phosphate in 200 mL of ultrapure water to obtain a pH of 7.4 and a concentration of 16 mM. The 6TG in PBS solution was prepared to obtain a maximum absorbance around 1 OD at the central wavelength of the pump beam (1.4 mM).

### 3.2. Linear Absorption

The linear absorption (LA) spectrum of each sample in PBS solution was measured using a JASCO Corp. V-570 (Jacso Inc., Easton, MD, USA) spectrometer at room temperature. The samples were placed in a 1 mm internal optical-path quartz cuvette. The spectra (Figure 1a) were background-corrected by subtracting the solvent spectrum measured under the same experimental conditions. The LA spectra were collected before and after each TAS experiment to assure that the sample had not degraded during the experiments.

### 3.3. Steady-State Photoluminescence

We acquired the steady-state emission spectra at room temperature using a Cary Eclipse spectrophotometer (Varian Inc., Palo Alto, CA, USA) with a lamp excitation. The spectra were measured at 1000 V of photomultiplier voltage with excitation and emission slit widths of 5 nm and averaging times of 0.9 s. The sample concentrations used were about 0.02 mM, and the contribution from the solvent solution was subtracted. After preparation, the sample was pumped with N_2_ to avoid O_2_ dissolved in the solution during the measurements that could lead to the formation of singlet oxygen and photoluminescence quenching.

### 3.4. Transient Absorption Spectroscopy

TAS measurements were carried out with a home-made setup [54] fed by an 800 nm Ti:Sapphire laser system (Libra, Coherent, Santa Clara, CA, USA), which delivers 100 fs pulses at a 1 kHz repetition rate. A fraction of the beam seeds a broadband, visible non-collinear optical parametric amplifier (NOPA), which was mixed with the fundamental wave in a 50 μm-thick Type II β-barium borate crystal to generate broadband UV pump pulses at 330 nm [55]. Probe pulses covering the 275–700 nm spectral range were obtained by white light generation in a 2 mm-thick CaF_2_ plate. The data were collected separated in two ranges: UV (275–340 nm) with thick CaF_2_ plate pumped at 400 nm and visible (330–650 nm) with thick CaF_2_ plate pumped at 800 nm. The two datasets were merged to obtain the data shown in Figure 2. The setup’s instrumental response function is estimated to be 20–30 fs (depending on the probe wavelength), and ultimately limits the precision of the determination of the faster time constant. For the measurements presented in this study, the pump energy was limited to 27 nJ (leading to a fluence of 340 μJ·cm^−2^) to avoid sample photodamage and solvated electrons generated by two-photon absorption from the solvent. A flowing jet configuration was employed for the sample that was flown at a typical rate of 40 rpm. The experiments were performed using pump and probe beams with parallel polarisations.

### 3.5. Global Analysis and Data Processing

The TAS datasets were subjected to a global analysis using the free software Glotaran [56,57]. A singular-value decomposition was first performed in order to find the number of linearly independent vectors (decay constants) significantly different from the noise. We found three dominant components and three residual components associated with the coherent artefact. A nonlinear, least-squares fit was then performed on the TAS map using a sequential kinetic model with three exponential functions. The time-dependent spectrum is thus represented by the function *ψ*(*λ*, *t*), and depends on the wavelengths of the probe and the delay between the pump and the probe:

(1) $$\psi (\lambda ,\;t)\;\propto \;\sum_{l=1}^{ncomp}(e^{klt}*IRF(t))EAS_{l}(\lambda )$$

where *k_l_* is the decay rate for each component, *IRF*(*t*) is the instrumental response function, and *EAS_l_*(*λ*) are the amplitudes of the exponential decays, known as evolution-associated spectra (EAS).

The analysis also takes into account the coherent artefact, the oscillatory time profile of which was modelled by a sequence of exponentials and the dispersion due to the chirp of the probe (modelled by a third-order polynomial).

At least three independent datasets (i.e., recorded on three different days) were used in the analysis, and all uncertainties are reported as twice the standard deviation of the average lifetime. The larger error in the determination of the faster time constant stems from the presence of the coherent artefact, which affects the dynamics in the first 100 fs.

## 4. Conclusions

From experimental data obtained by TAS with sub-20 fs pump pulses and broadband probing in a joint UV and visible window, combined with global analysis, we demonstrate the involvement of a dark singlet state (S_1_(nπ*)) in the decay mechanism of 6TG, which acts as a doorway to the triplet manifold. The ultrafast data are supported by theoretical studies and establish that the major decay pathway in 6TG to the triplet manifold is as follows: S_2_(ππ*)→S_1_(nπ*)→T_1_(ππ*).

## Figures and Tables

**Table 1:** Emission energies and oscillator strengths (f) calculated in-vacuum for the S_2_(ππ*) state at the Frank–Condon (FC) [43] at three single points (P_1_, P_2_, and P_3_), along the potential energy surface of S_2_(ππ*) before reaching the S_2_(ππ*)/S_1_(nπ*) CI and for the S_1_(ππ*) and S_2_(nπ*) minima [43].

State Character	Vertical Emission Energy eV (nm)	Adiabatic Emission Energy eV (nm)	f
FC→S_2_(ππ*)	4.00 (311)		0.187
P_1_ S_2_(ππ*)→S_0_(ππ*)	3.16 (393)	3.87 (320)	0.101
P_2_ S_2_(ππ*)→S_0_(ππ*)	3.04 (408)	3.87 (320)	0.095
P_3_ S_2_(ππ*)→S_0_(ππ*)	2.86 (434)	3.78 (328)	0.061
S_1_(ππ*)_min_→S_0_(ππ*)	2.29 (541)	3.79 (328)	0.024
S_1_(*n*π*)_min_→S_0_(ππ*)	2.14 (580)	3.18 (392)	0.001

## Data Availability

Data are available from the authors upon reasonable request.

## Supplementary Materials

The following supporting information can be downloaded online, Figure S1: Minimum energy path from which the single points calculations were extracted. CASSCF (14,12)/ANO-L level of theory.; Table S1: XYZ coordinates.

## Sample Availability

Not available.
