# Supplementary material for: Ultrafast Excited-State Decay Mechanisms of 6-Thioguanine Followed by Sub-20 fs UV Transient Absorption Spectroscopy
Source: Molecules. 2022 Feb 10;27(4):1200. doi: 10.3390/molecules27041200 (PMC8878119; doi:10.3390/molecules27041200)
Supplement: Supplementary file 1 [file molecules-27-01200-s001.zip › molecules-1544201-supplementary.pdf]

Ultrafast excited-state decay mechanisms of 6-thioguanine followed by sub-20 fs UV transient absorption spectroscopy

Danielle C. Teles-Ferreira[1], Cristian Manzoni[2], Lara Martínez-Fernández[3], Giulio Cerullo[2,4], Ana Maria de Paula[5] and Rocío Borrego-Varillas[2,*]

[1] Instituto Federal de Minas Gerais, Campus Ouro Preto, Ouro Preto 35400-000, MG, Brazil; danielle.teles@ifmg.edu.br

[2] IFN-CNR, Piazza Leonardo da Vinci 32, I-20133 Milano, Italy; cristian.manzoni@polimi.it (C.M) ; rocio.borrego@polimi.it (R.B.V)

[3] Departamento de Química, Facultad de Ciencias and Institute for Advanced Research in Chemistry (IADCHEM), Universidad Autónoma de Madrid, Campus de Excelencia UAM-CSIC, Cantoblanco, 28049 Madrid, Spain; lara.martinez@uam.es

[4] Dipartimento di Fisica, Politecnico di Milano, Piazza Leonardo da Vinci 32, I-20133 Milano, Italy; giulio.cerullo@polimi.it

[5] Departamento de Física, Universidade Federal de Minas Gerais, Belo Horizonte 31270-901, MG, Brazil; ana@fisica.ufmg.br

* Correspondence: rocio.borrego@polimi.it

Supplementary material

A. Computational Details
B. Computational Results
C. XYZ Coordinates

A. Computational Details

The same protocol as described in [1] has been applied to compute the adiabatic, emission energies and oscillator strengths at different points along the Minimum Energy Path (MEP) depicted in Figure S1. In short, MS-CASPT2//CASSCF (14,12)/ANO-L [2–5]calculations were carried out on top of P1, P2 and P3 geometries (section C) that were extracted from the CASSCF (14,12)/ANO-L MEP. This MEP was performed staring from the Franck-Condon region (taken from [1] and relaxing the S_2 state. The OpenMolcas [6]program was used throughout this study.

B. Computational Results

Figure S1. Minimum energy path from which the single points calculations were extracted. CASSCF (14,12)/ANO-L level of theory.

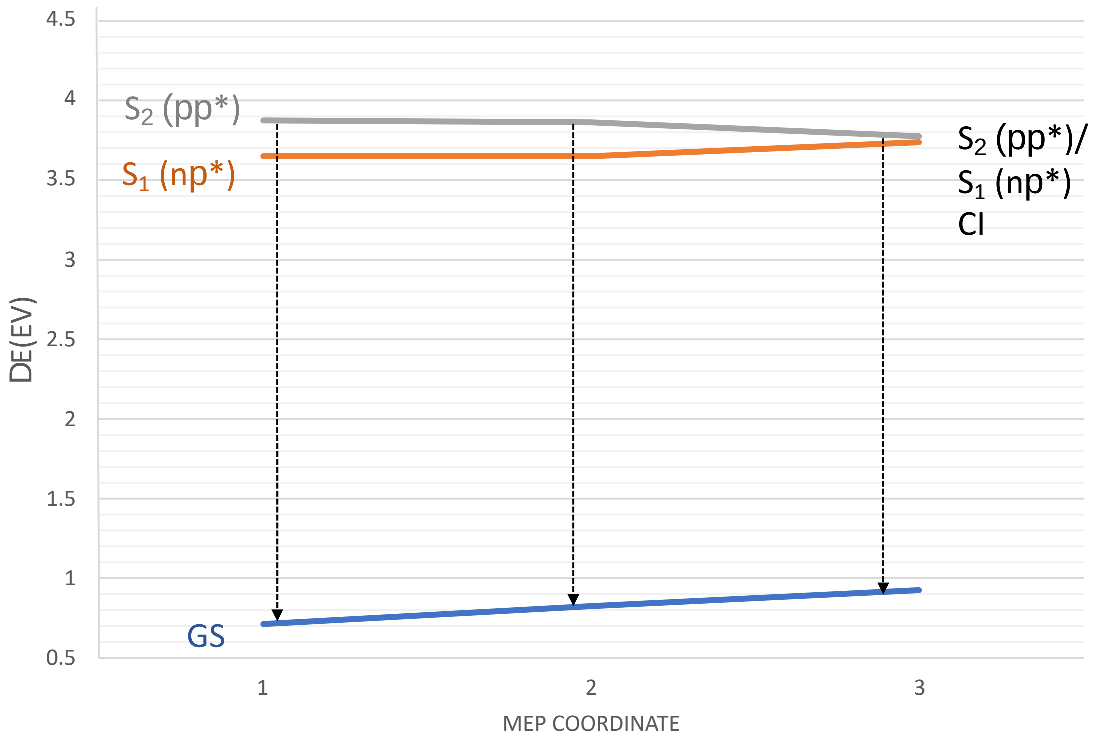

C. XYZ coordinates

P1

H	-2.360193	0.789528	-0.115714
H	-2.743765	-2.672667	0.050775
H	-3.482201	-1.281024	0.498386
H	2.284988	-2.546938	-0.065828
H	3.856002	-0.569188	-0.015149
C	-1.539482	-1.089427	0.011595
C	-0.326720	1.005936	0.015688
C	0.810213	0.293398	0.045372
C	0.683976	-1.168169	-0.004625
C	2.793158	-0.475941	-0.000920
N	-1.517170	0.295255	0.032574
N	-2.788828	-1.690595	-0.087518
N	-0.412327	-1.836970	-0.008481
N	2.163916	0.639520	0.039087
N	1.989543	-1.606953	-0.026459
S	-0.511718	2.768456	-0.025555

P2

H	-2.331997	0.799672	-0.175655
H	-2.721443	-2.667917	-0.003111
H	-3.456004	-1.329140	0.554486
H	2.298655	-2.566466	-0.063618
H	3.867954	-0.593877	-0.012627
C	-1.532620	-1.088694	0.048816
C	-0.309577	1.003128	0.020648
C	0.824103	0.278271	0.034076
C	0.693107	-1.176354	-0.000806
C	2.804255	-0.501727	-0.001121
N	-1.505085	0.297533	0.032417
N	-2.788213	-1.680207	-0.098251
N	-0.413499	-1.834990	-0.007902
N	2.181939	0.618287	0.033890
N	1.997297	-1.628198	-0.023994
S	-0.542649	2.798557	-0.029576

P3

H	-2.311533	0.777313	-0.279123
H	-2.683635	-2.660775	-0.159336
H	-3.439352	-1.446939	0.591842
H	2.279531	-2.565352	-0.082177
H	3.869497	-0.599913	-0.005643
C	-1.534307	-1.084260	0.126943
C	-0.314565	1.019962	0.031983
C	0.823851	0.289924	0.036529
C	0.688597	-1.169025	-0.017463
C	2.805589	-0.502646	-0.000927
N	-1.500390	0.304291	0.038607
N	-2.782406	-1.670309	-0.126665
N	-0.398276	-1.838878	-0.020256
N	2.184669	0.618824	0.040836
N	1.989190	-1.623521	-0.037726
S	-0.566697	2.787968	-0.026616

References

1. Martínez-Fernández, L.; González, L.; Corral, I. An ab initio mechanism for efficient population of triplet states in cytotoxic sulfur substituted DNA bases: The case of 6-thioguanine. *Chem. Commun.* **2012**, *48*, 2134–2136, doi:10.1039/c2cc15775f.
2. Andersson, K.; Malmqvist, P.Å.; Roos, B.O. Second-order perturbation theory with a complete active space self-consistent field reference function. *J. Chem. Phys.* **1992**, *96*, 1218–1226, doi:10.1063/1.462209.
3. Roos, B.O.; Andersson, K. Multiconfigurational perturbation theory with level shift - the Cr2 potential revisited. *Chem. Phys. Lett.* **1995**, *245*, 215–223, doi:10.1016/0009-2614(95)01010-7.
4. Finley, J.; Malmqvist, P.Å.; Roos, B.O.; Serrano-Andrés, L. The multi-state CASPT2 method. *Chem. Phys. Lett.* **1998**, *288*, 299–306, doi:10.1016/S0009-2614(98)00252-8.
5. Pou-Amérigo, R.; Merchán, M.; Nebot-Gil, I.; Widmark, P.O.; Roos, B.O. Density matrix averaged atomic natural orbital (ANO) basis sets for correlated molecular wave functions - III. First row transition metal atoms. *Theor. Chim. Acta* **1995**, *92*, 149–181, doi:10.1007/BF01114922.
6. Fdez. Galván, I.; Vacher, M.; Alavi, A.; Angeli, C.; Aquilante, F.; Autschbach, J.; Bao, J.J.; Bokarev, S.I.; Bogdanov, N.A.; Carlson, R.K.; et al. OpenMolcas: From Source Code to Insight. *J. Chem. Theory Comput.* **2019**, *15*, 5925–5964, doi:10.1021/acs.jctc.9b00532.
